# The Role of Egg Yolk in Modulating the Virulence of *Salmonella Enterica* Serovar Enteritidis

**DOI:** 10.3389/fcimb.2022.903979

**Published:** 2022-06-14

**Authors:** Yumin Xu, Ahmed G. Abdelhamid, Anice Sabag-Daigle, Michael G. Sovic, Brian M.M. Ahmer, Ahmed E. Yousef

**Affiliations:** ^1^ Department of Food Science and Technology, The Ohio State University, Columbus, OH, United States; ^2^ Botany and Microbiology Department, Faculty of Science, Benha University, Benha, Egypt; ^3^ Department of Microbial Infection and Immunity, The Ohio State University, Columbus, OH, United States; ^4^ Center for Applied Plant Sciences, The Ohio State University, Columbus, OH, United States; ^5^ Department of Microbiology, The Ohio State University, Columbus, OH, United States

**Keywords:** *Salmonella* Enteritidis, virulence, transcriptomic analysis, mouse model, stress response, RNA sequencing

## Abstract

Contribution of food vehicles to pathogenicity of disease-causing microorganisms is an important but overlooked research field. The current study was initiated to reveal the relationship between virulence of *Salmonella enterica* serovar Enteritidis and egg yolk as a hosting medium. Mice were orally challenged with *Salmonella* Enteritidis cultured in egg yolk or tryptic soy broth (TSB). Additionally, mice were challenged with *Salmonella* Enteritidis cultured in TSB, followed by administration of sterile egg yolk, to discern the difference between pre-growth of the pathogen and its mere presence in egg yolk during infection. The pathogen’s Lethal dose 50 (LD_50_) was the lowest when grown in yolk (2.8×10^2^ CFU), compared to 1.1×10^3^ CFU in TSB, and 4.6×10^3^ CFU in TSB followed by administration of sterile yolk. Additionally, mice that orally received *Salmonella* Enteritidis grown in egg yolk expressed a high death rate. These findings were supported by transcriptional analysis results. Expression of promoters of virulence-related genes (*sopB* and *sseA*) in genetically modified *Salmonella* Enteritidis reporter strains was significantly higher (*p* < 0.05) when the bacterium was grown in the yolk, compared to that grown in TSB. Sequencing of RNA (RNA-seq) revealed 204 differentially transcribed genes in *Salmonella* Enteritidis grown in yolk *vs*. TSB. Yolk-grown *Salmonella* Enteritidis exhibited upregulated virulence pathways, including type III secretion systems, epithelial cell invasion, and infection processes; these observations were confirmed by RT-qPCR results. The transcriptomic analysis suggested that upregulation of virulence machinery of *Salmonella* Enteritidis grown in egg yolk was related to increased iron uptake, biotin utilization, flagellar biosynthesis, and export of virulence proteins encoded on *Salmonella* pathogenicity island 1, 2, 4, and 5. These biological responses may have acted in concert to increase the virulence of *Salmonella* infection in mice. In conclusion, growth in egg yolk enhanced *Salmonella* Enteritidis virulence, indicating the significance of this food vehicle to the risk assessment of salmonellosis.

## 1 Introduction

Shell eggs are frequently associated with salmonellosis (CDC, 2010; CDC, 2016; CDC, 2018a; CDC, 2018b) and *Salmonella enterica* serovar Enteritidis is often linked to these outbreaks (Braden, 2006). It was estimated that among approximately 65 billion eggs produced annually in the United States, 3.25 million (0.005%) are contaminated with *Salmonella* Enteritidis (Schroeder et al., 2005). Shell eggs become contaminated with *S. enterica* through a horizontal or vertical transmission route (Howard et al., 2012). During horizontal contamination, the pathogen is gradually internalized from the surface of the eggshell, whereas in vertical contamination, pathogen cells are transferred from the infected reproductive organs into the forming egg. In both transmission routes, *S. enterica* can migrate into the nutrient-rich egg yolk and proliferate into a large population (Gantois et al., 2009).

Fitness of hens and their eggs as hosts for *Salmonella* serovars, prior to human infection, has been identified in several studies. *Salmonella* Enteritidis seems to have an exceptional genetic advantage to colonize a hen’s oviduct during systemic infection, allowing the pathogen’s access to the egg through the vertical transmission route (Guard-Petter et al., 1997; Parker et al., 2001; Parker et al., 2002; Lu et al., 2003; Buck J et al., 2004; Morales et al., 2005; Mizumoto et al., 2005; Gantois et al., 2008; Shah et al., 2017). Wang et al. (2018) also confirmed that genetic repertoires in two *Salmonella* Enteritidis strains isolated from poultry resulted in notable differences in their survivability in egg white. However, Shah (Khan et al., 2021) showed that genetically homogeneous *Salmonella* Enteritidis strains could vary phenotypically in their virulence under the same experimental conditions, and the researchers concluded this was caused by differences in the strains’ ability to express virulence and stress response genes.

Egg yolk may serve as an ideal medium for *Salmonella* Enteritidis prior to infection of a susceptible host. Although Baron et al. (2017) concluded that egg white did not induce the expression of *Salmonella* Enteritidis virulence, a recent study provided evidence that *Salmonella* Typhimurium in egg yolk, but not in egg white or on egg shell, showed increased transcription of virulence genes (Khan et al., 2021). Other researchers demonstrated that *Salmonella* Enteritidis grown in egg yolk displayed elevated ability to colonize intestines and express disease markers in a mouse model of human colitis (Moreau et al., 2016); however, the mechanism behind this increased pathogenicity is still unclear.

Multiple studies demonstrated the impact of food on the infectious ability of *Salmonella* serovars. For instance, food components may contribute to bacterial survival against the first line of defense in the human gastrointestinal tract; stomach acidity (Waterman and Small, 1998; Koseki et al., 2011; Aviles et al., 2013). Additionally, stress exerted by certain food components onto pathogens could increase their survival in the gastrointestinal tract and virulence (Yuk and Schneider, 2006; Oliveira et al., 2011; Aviles et al., 2013). Considering that several salmonellosis outbreaks were associated with consumption of egg, it is necessary to investigate whether egg components, particularly yolk, induce virulence in *Salmonella* Enteritidis. Additionally, exploring the underlying mechanism by which egg yolk shapes the pathogenicity of this bacterium is imperative.

Epidemiological investigations predicted a wide range of infectious doses (1.1 ×10^1^ to 7.3 × 10^5^ CFU) of *Salmonella* Enteritidis in egg and egg-related products (Levy et al., 1996; WHO/FOA, 2002; Teunis et al., 2010). Currently, the Lethal dose 50 (LD_50_) of *Salmonella* Enteritidis in egg yolk is only broadly defined. Considering that the infectious dose is one of the primary factors to consider in risk assessment of foodborne pathogens, it is critical to determine with greater accuracy the LD_50_ of *Salmonella* Enteritidis in the egg environment. Therefore, the current study was initiated to determine *Salmonella* Enteritidis LD_50_ in mice fed *Salmonella* Enteritidis that was cultured in different media, including egg yolk. It should be noted that death of the animal is defined here as meeting the early removal criteria (ERC) for removal of animals from the study by humane euthanasia. Molecular mechanisms underlying differential pathogenicity were then evaluated by assessing transcriptional changes in *Salmonella* Enteritidis virulence genes when the pathogen was grown in egg yolk.

## 2 Material and Methods

### 2.1 Ethics Statement

The animal study was reviewed and approved by the Ohio State University Institutional Animal Care and Use Committee (Protocol OSU 2009A0035). Mice were carefully inspected daily and once their body weight loss or disease symptoms met the early removal criteria (ERC), they were removed immediately. Carbon dioxide asphyxiation (replacement of air in a cage with 100% CO2) was then applied followed by cervical dislocation (applying pressure to the neck and disarticulating the cervical vertebrae from the skull) was used for euthanizing the mice.

### 2.2 Bacterial Strains

*Salmonella* Enteritidis ODA 99‐30581‐13 (Perry et al., 2008; Perry et al., 2011; Perry and Yousef, 2013), isolated originally from shell eggs and provided by the Ohio Department of Agriculture (Reynoldsburg, OH, USA), were used in the current study. Two reporter strains were generated in this study; these were derivatives of *Salmonella* Enteritidis ODA 99‐30581‐13. Two strains, *Salmonella* Typhimurium pBA409 and pRG49, were used for plasmids retention. All strains were streaked from frozen stocks, held at -80°C in tryptic soy broth (TSB; Bacton, Dickson and Company, Franklin Lakes, NJ, USA) containing 15% glycerol (Sigma-Aldrich, Burlington, MA, USA), onto tryptic soy agar (TSA; Bacton, Dickson and Company). Inoculated agar plates were incubated at 37°C for 18 h and single colonies were sub-cultured onto fresh TSA plates, which were subsequently incubated at 37°C for 18 h. Single colonies from the sub-cultures were inoculated into TSB tubes (5 mL each) and incubated at 37°C for 18 h. Reporter strains were prepared under similar conditions except that all media were supplemented with 10 µg/mL tetracycline (Sigma-Aldrich).

### 2.3 *Salmonella* Inoculation in Yolk and Microbiological Media

Unfertilized washed Grade AA large shell eggs were obtained, 5 to 10 days after laying, from *Salmonella*-free flock on the farms of Hertzfeld farms (Grand Rapids, OH, USA). The eggs were stored at 4°C and used in experiments within a week of storage. Egg surfaces were decontaminated by submerging intact eggs in ethanol (70% v/v) for 1 min. Eggs were removed and excess ethanol was flamed. The yolk was separated from the egg using a sterile stainless-steel yolk separator and placed in a homogenizer bag (Fisher Scientific, Fair Lawn, NJ, USA). The sterility of the yolk was ensured by spreading 100 µL of the yolk onto a TSA plate which was incubated at 37°C for 24 h. The yolk of three eggs was collected and homogenized for 1 min before 15 mL (approximately equivalent to the volume of the yolk of one egg) were transferred into the sterile 50-mL conical centrifuge tube. Meanwhile, 15 mL of TSB were also transferred to another 50-mL conical centrifuge tube. *Salmonella* Enteritidis cell suspension was prepared by separating the cells in the TSB overnight culture by centrifugation and washing the resulting pellet three times using sterile saline (0.85% NaCl) solution (Fisher Scientific). The resulting cell suspension was diluted 1:10,000 in the saline solution before 10 µL of which was added to the yolk or TSB (final population of approximately 10^3^ CFU per 15 mL egg yolk or TSB). After proper mixing, the inoculated media were incubated at 30°C before use in further experiments; this incubation temperature mimics the worst-case-scenario for storage of naturally-contaminated eggs (Perry and Yousef, 2013).

### 2.4 LD_50_ and Survival Curves of Infected Mice

To determine if presence of *Salmonella* Enteritidis in egg yolk increases its virulence, an *in vivo* study was conducted using female C57BL/6 mice obtained at 6 to 8 weeks of age (The Jackson Laboratory, Bar Harbor, ME, USA). The study was performed at the Veterinary Medicine Complex at The Ohio State University abiding by protocols approved by The Ohio State University Institutional Animal Care and Use Committee (IACUC; OSU 2009A0035). The mice were inoculated *via* intragastric gavage and received either *Salmonella* grown in TSB (Bacton, Dickson and Company), *Salmonella* grown in yolk, or *Salmonella* grown in TSB followed by sterile yolk; within each treatment group, each cage of five mice was challenged with one of the 10-fold dilutions of *Salmonella* from 10^7^ CFU to 10^2^ CFU, the same growth medium was used as diluent. Control groups included mice treated with sterile TSB, sterile yolk and sterile TSB followed by sterile yolk. Body weight and ERC were closely monitored to determine euthanizing point. The experiment was repeated twice.

### 2.5 Genetic Transformation

#### 2.5.1 Construction of *Salmonella sopB* and *sseA* Reporter Strains

Plasmids pBA409 and pRG49 are derived from pSB401 (Winson et al., 1998) which encodes p15A, a tetracycline resistance marker and a promoterless *luxCDABE* operon. For pBA409, the promoter fragment of pSB401 was replaced with the regulatory region of the gene encoding inositol phosphate phosphatase from *Salmonella* Typhimurium SPI-1 effector, *sopB* (Goodier and Ahmer, 2001). To construct pRG49, the promoter region of the *Salmonella* Typhimurium SPI-2 gene, *sseA*, was amplified with PCR (using primers CGGCAAGTTACAGGATCCGCAGCAA and CTGACGGTATCTCCACCGGGGCTTG), cloned into pCR-blunt II Topo, removed with EcoRI, and ligated to pSB401 that had also been digested with EcoRI, thus replacing the original promoter fragment. pBA409 and pRG49 were then electroporated into *Salmonella* Enteritidis ODA 99‐30581‐13 (**Table 1**).

**Table 1:** Characteristics of *Salmonella* Enteritidis strains constructs in the current study.

Strain	Source or description	GenBank accession/[Reference]
*Salmonella* Enteritidis ODA 99‐30581‐13	Isolated from chicken egg	NZ_JACTGY010000040.1/ (95–97)
*Salmonella* Typhimurium pBA409	Containing plasmid *sopB*::luxCDABE	(Goodier and Ahmer, 2001)
*Salmonella* Typhimurium pRG49	Containing plasmid *sseA*::luxCDABE	(Current study)

#### 2.5.2 Luciferase Activity

Egg yolk was inoculated with reporter *Salmonella* strains as previously mentioned. Luciferase activity of reporter strains was measured at 10, 12, 14, 16, 18, 20, and 24 h during *Salmonella* growth in egg yolk at 37°C to indicate the expression of the virulence genes. At each sampling point, aliquots (200 µL) were distributed into 96-well microplates (Corning, Fisher Scientific) and the luminescence of each well was measured using a multilabel counter (Wallac Victor 3; PerkinElmer Life and Analytical Sciences, Shelton, CT, USA). The bacterial population was also determined at each sample by spread plating onto TSA supplemented with 10 µg/mL of tetracycline and the luciferase activity was normalized against *Salmonella* population. Each experiment was repeated three times.

### 2.6 Transcriptomic Analysis Using RNA Sequencing

#### 2.6.1 Determining *Salmonella* Growth Curve and Stage of RNA Extraction

Inoculated TSB or yolk was sampled after 0, 1, 2, 4, 7, 10, 13, 16, 24, and 48 h incubation at 30°C and the *Salmonella* Enteritidis population at each time point was measured by spread-plating technique using TSA medium. The growth curves, from three independent repetition, were fit into the modified Gompertz model (Juneja et al., 2009),


$$N=a+\left(b-a\right)e^{-e^{-c\left(t-d\right)}}$$

where “N” is *Salmonella* Enteritidis population (log CFU/mL), “a” is the lower asymptote (log CFU/mL), “b” is the upper asymptote (log CFU/mL), “c” is the growth rate (log CFU/mL/h), “d” is the inflection point (h), and “t” is time (h). The model’s mathematical parameters (a, b, c, and d) were determined using statistical software (JMP Pro 14; SAS Institute Inc., Cary, NC). Late log phase, when the growth of *Salmonella* Enteritidis in the TSB reached 1.5 × 10^8^ CFU/mL, was selected for RNA extraction. By plugging the desired final population into the *Salmonella* growth curve model, the time to reach that population could be calculated to determine the RNA extraction point for yolk and TSB.

#### 2.6.2 RNA Extraction and Quality Assessment

To facilitate RNA extraction, *Salmonella* cells were captured from inoculated and incubated egg yolk and TSB using magnetic beads coated with *Salmonella*-antibody (Dynabeads anti-*Salmonella*; Life Technology, Carlsbad, CA, USA). Magnetic beads (200 µL) were added to 1 ml of egg yolk that was diluted with 9 mL of saline. The mixture was homogenized on the sample mixer (Dynabeads Rotator Mixer 947-01; Invitrogen, Carlsbad, CA, USA) for 15 min. After the beads were recovered by attaching the tube to the magnetic plate (Dynal Magnetic Particle Concentrator-6 120-02D; Invitrogen) for 3 min, the supernatant was discarded, and the beads were resuspended in saline. This washing step was repeated two additional times before the beads were resuspended in 100 µL of saline which was later transferred to the 2-mL screw-capped tube (Fisher Scientific) containing a mixture of 400 mg of 0.1 mm and 400 mg of 0.5-mm glass beads each (BioSpec Products, Inc., Bartlesville, OK, USA) and 600 µL of Buffer RLT (RNeasy mini kit; Qiagen, Germantown, MD, USA) supplemented with 6 µL of β-mercaptoethanol (Bio-Rad, Hercules, CA, USA). The mixture was treated in the homogenizer (4-Place Mini Bead Mill Homogenizer, VWR, Montigny le Bretonneux, France) at 5 m/s for three 60-s intervals to release the RNA from the bacterial cells. The tubes were cooled on ice for 1 min between homogenization intervals. The homogenized samples were centrifuged (5415R centrifuge; Eppendorf, Hauppauge, NY, USA) at 12,000 × *g* and 4°C for 5 min and the RNA was purified from the supernatant using a purification kit (RNeasy mini; Qiagen) according to manufacturer’s instructions. The RNA quality and quantity were determined using a spectrophotometer (NanoVue; GE Healthcare Life Sciences, Buckinghamshire, UK). The ratio of 260 and 280 was determined to assess the RNA purity. The integrity of RNA was determined using bioanalyzer (Agilent 2100, Agilent Technologies, Santa Clara, CA, USA) and only RNA preparations with integrity number greater than 7 were used for subsequent experiments.

#### 2.6.3 Library Construction and RNA Sequencing

The library construction and sequencing were conducted by Novogene Co. (Davis, CA, USA) from 250 ng input of each RNA sample. To remove rRNA from total RNA, a commercial kit (Ribo-zero rRNA removal kit (Bacteria); Illumina, San Diego, CA, USA) was used according to the manufacturer’s protocol. The strand-specific mRNA library was subsequently constructed with the NEBNext Ultra Directional RNA Library Prep Kit (New England BioLabs Inc., MA, USA). In brief, enriched mRNA was fragmented using divalent cations (RNA fragmentation reagent, Life Technology, CA, USA) in buffer (NEBNext First Strand Synthesis Reaction Buffer; New England BioLabs Inc.). First strand cDNA was synthesized using random hexamer primer and RNA-dependent DNA polymerase, Moloney Murine Leukemia Virus Reverse Transcriptase (M-MuLV), prior to second strand cDNA synthesis, during which dUTP, dATP, dCTP, and dGTP were applied. The exonuclease and polymerase were used to repair the DNA overhangs and adenylate the 3’ ends of the DNA fragments. The library fragments were multiplexed using the index primer kit (NEBNext Multiplex Oligos for Illumina kit; New England BioLabs Inc.). Hairpin loop structured NEBNext Adapters were ligated to the DNA fragments, which were cleaned up by bead-based AMPure XP purification system (Beckman Coulter, Beverly, USA) to select for cDNA fragments that were 150 to 200 bp in length. Uracil excision through uracil-specific excision enzyme (USER, New England BioLabs Inc.) and PCR enrichment through DNA polymerase (Phusion™ High-Fidelity DNA polymerase, Fisher Scientific), universal PCR primers and Index (X) Primer were performed on the purified cDNA fragments to create indexed libraries. The barcoded cDNA fragments were purified again using AMPure XP system before the library quality was assessed on the Agilent Bioanalyzer 2100 (Agilent Technologies). The libraries were clustered on the cBot Cluster Generation System using HiSeq PE Cluster Kit cBot-HS (Illumina, CA, USA) based on manufacturer’s instruction and sequenced on the Illumina HiSeq 2500 platform (Illumina; paired-end, 150 bp per read). Three biological repeats were analyzed for both the control group and the treatment group (3 × 2) rendering six samples in total.

#### 2.6.4 Read Alignment and Analysis of Gene Transcription Data

Initial sequencing quality was assessed with metrics including GC content and the proportion of reads with average base call scores exceeding quality score of 20 (Q20) and Q30. Adaptors were trimmed and low quality base calls were removed using Trimmomatic (Bolger et al., 2014) with parameters ILLUMINACLIP:[*adaptor.fa*]:2:30:10 LEADING:3 TRAILING:3 SLIDINGWINDOW:4:15 MINLEN:36. Trimmed fastq files were aligned to *Salmonella* Enteritidis strain P125109 using Subread 1.5.0 (Liao et al., 2013) and aligned bam files were sorted and duplicate reads marked using Picard 2.3.0 (Picard Tools - By Broad Institute). Reads were then counted using FeatureCounts (Liao et al., 2014) and analyzed for differential expression using DESeq2 1.24.0 (Love et al., 2014). Differentially expressed genes (DEGs) were identified as those with padj < 0.05. DEGs were analyzed against Gene Ontology (GO) and Kyoto Encyclopedia of Genes and Genomes (KEGG) database to annotate their main biological functions and pathways, and the Search Tool for Retrieval of Interacting Genes/proteins (STRING) analysis (Szklarczyk et al., 2019) was performed to identify connections between different pathways. Gene Set Enrichment Analysis (GSEA (Mootha et al., 2003; Subramanian et al., 2005),) was also conducted to identify significantly up- or downregulated pathways from a ranked list of all sampled genes. Gene sets (pathways) analyzed included KEGG pathways listed under the *Salmonella* Enteritidis P125109 (KEGG genome ID T00776) that contain > 5 genes.

#### 2.6.5 Preparation of cDNA for Reverse Transcription Quantitative PCR (RT-qPCR) Analysis

DNA was removed from the extracted RNA by DNase of a commercial kit (Turbo DNase kit, Invitrogen, Vilnius, Lithuania) following the kit manufacturer’s instructions. The DNase-treated samples were measured for final RNA concentration (NanoVue spectrophotomer) and stored in the -80°C freezer until further use. The RNA was transcribed to cDNA using cDNA reverse transcription kit (High-Capacity cDNA, Applied Biosystems, Vilnius, Lithuania) according to manufacturer’s instructions. The cDNA synthesis was performed using PCR thermocycler (GeneAmp PCR System 2400, Applied Biosystems, Waltham, MA, USA) set to run the following sequence: 25°C for 10 min, 37°C for 120 min, and 85°C for 5 min. The cDNA was diluted using 80 µL sterile double-ionized water and stored at -20°C until use.

#### 2.6.6 RT-qPCR Analysis

RT-qPCR was performed to assess the differential transcription of selected genes, spanning *Salmonella* pathogenicity islands (SPI) 1 to 5, using the specific primers reported in **Table 2**. A comprehensive panel of virulence genes (up to 23), with putative functions as regulators for SPIs and effectors or secretions apparatus, were investigated. Before RT-qPCR, the primers were tested on conventional PCR to ensure their specificity and to optimize the annealing temperature (Mundi et al., 2013). For each 20-µL reaction, 5 μL of cDNA was added to the 10 µl SYBR Select PCR master mix (Applied Biosystems), 500 nM of each of the forward and reverse primers (**Table 2**; Millipore Sigma, Danvers, Massachusetts) and proper volume of water. Using real-time PCR system (7900HT; Applied Biosystems), PCR cycles were conducted in sequence as following: 50°C for 2 min (stage 1); 95°C for 7 min (stage 2); 95°C for 15 s, 60°C for 1 min (stage 3, repeated 40 times). Each reaction was triplicated within each RT-qPCR run and the RT-qPCR experiment for each reaction was independently repeated three times.

**Table 2:** Primers used for RT-qPCR to detect the expression of virulence genes of *Salmonella* Enteritidis grown in egg yolk and tryptic soy broth.

Gene	Gene function	Forward primer	Reverse primer	Reference
*gapA*	Housekeeping gene used for normalization	GGTGTTGACGTAGTGGCTGAA	AGCGTTGGAAACGATGTCCTG	(Finn et al., 2013)
*hilC*	Regulatory genes for SPI-1	CCAGTTTTCGCTTCAGACTTGA	CACCCGCAAATGGTCACA	(Kollanoor Johny et al., 2017)
*hilD*		TGGCGCTCTCTATGCACTTA	AACGCCGTTTTCAGATGTTC	(Weir et al., 2008)
*invF*		ATGATTAACGGCTAATTGGGTGA	CGGAAAAGCGAAGAGTGAATTAC	(Campos-Galvao et al., 2016)
*sipA*	SPI-1 effector genes involved in lipid metabolism	TCTGCTTTTTTCCCACCATCA	AGATAAACTGCCTGACCCTAAAATTC	(Kollanoor Johny et al., 2017)
*sopB*		GCGTCAATTTCATGGGCTAAC	GGCGGCGAACCCTATAAACT	(Upadhyaya et al., 2013)
*sipB*		GCCACTGCTGAATCTGATCCA	CGAGGCGCTTGCTGATTT	(Upadhyaya et al., 2013)
*sopE*	Important SPI-1 effector genes	CAACACACTTTCACCGAGGAAG	GGTCTGGCTGGCGTATGC	(Choi et al., 2007)
*sigD*		AACCGTTCTGGGTAAACAAGAC	GGTCCGCTTTAACTTTGGCTAAC	(Choi et al., 2007)
*ssrA*	Regulatory genes for SPI-2	CGAGTATGGCTGGATCAAAACA	TGTACGTATTTTTTGCGGGATGT	(Upadhyaya et al., 2013)
*ssrB*		CGCAGGTGCTAATGGCTATG	TTGCAATGCCGCTAACAGA	(Li et al., 2015)
*sseJ*	SPI-2 effector genes involved in lipid metabolism	TATTACGAGACTGCCGATGC	GCCCGTGGTGAGTATAAGGGT	(Lee et al., 2012)
*sseL*		CCCAACGGCTGTGGTCTATT	TATTTGTCGCCGGGTTTGGG	This study
*sopD*	Important SPI-2 effector genes	ACTCCATCATTCACGGGCAG	TTGCTTTGGCTGATCACCGT	
*marT*	Regulatory genes for SPI-3	TACCGACAGCAAATGTCCCG	AGATTGAGCCAGGTGTGAGC	
*mgtB*	Important SPI-3 effector genes	ACAACCAGTGTCTGCGAGAG	ACCTTTGCGCTGATGTGGTA	
*mgtC*		CGAACCTCGCTTTCATCTTCTT	CCGCCGAGGGAGAAAAAC	(Upadhyaya et al., 2013)
*siiC*	Important SPI-4 effector genes	AGCTACAGCAACTCTCATTGATTT	CCCTGACCATGAACCACTGAA	This study
*siiE*		AGAATCGCCTCGCTTACTCG	CAGAACCTGCCACCATAGCA	
*pipB*	Important SPI-5 effector genes	GCTCCTGTTAATGATTTCGCTAAAG	GCTCAGACTTAACTGACACCAAACTAA	(Upadhyaya et al., 2013)
*spvR*	Regulatory genes for *Salmonella* virulence plasmid	AAGGGCTGGCGATGTTACTC	GGTGTCTCCCGTTTCTTGGT	This study
*spvA*	Important effector genes on *Salmonella* virulence plasmid	CGGTATTCAGGGACAGGCAG	ATCTTCCAGCGACACATCGG	
*spvB*		GGGTGGGCAACAGCAA	GCAGGATGCCGTTACTGTCA	(Upadhyaya et al., 2013)
*flhD*	Regulatory gene for flagellar biosynthesis	CGTTTGATCGTCCAGGACAA	TGTTTGCCATCTCTTCGTTGAT	(Kollanoor Johny et al., 2017)

### 2.7 Statistical Analysis

Survival curves were generated by a statistical software (GraphPad Prism 9.0.0; GraphPad Software, San Diego, CA, USA) and the data from two repeats were combined and compared using Gehan-Breslow-Wilcoxon test, which was adjusted by Tukey-Kramer test. LD_50_ was calculated by combining the data from two repeats and fitting the dose-response curve. The fit of all three LD_50_ curves was compared as a whole model by GraphPad Prism 9.0.0 using a least squares regression model and significance was determined at a *p-*value of < 0.05. The parallelism F-test and parallelism Chi-square test were used to compare the growth model of *Salmonella* in the yolk and TSB, using a statistical software (JMP Pro 14). To demonstrate the transcription level of virulence genes, luminescence was normalized against the bacterial population and normalized luminescence levels were statistically compared using two-way ANOVA (two factors: time and treatment). RT-qPCR data were analyzed using ΔΔCt method (Pfaffl, 2001) using *gapA* as the housekeeping gene to compare the expression level of *Salmonella* virulence genes in yolk compared to TSB. Data with ≥ 2-fold changes were considered as significant. A cutoff at 5% of false discovery rate (FDR) was used for determining significance of KEGG, GO, and STRING analysis and for GSEA analysis, a cutoff at 0.05 of normalized *p* value was used. Except for the RT-qPCR data and differentially expressed gene analysis, all statistical analysis with a *p-*value of < 0.05 was considered as significant.

## 3 Results

### 3.1 Growth in Egg Yolk Changes *Salmonella* Enteritidis Virulence in Mice

**Figure 1:**
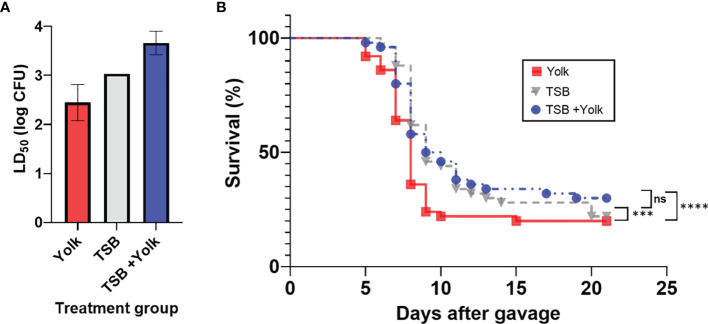
Pathogenicity of *Salmonella* Enteritidis to mice that were fed, through oral gavage, the pathogen that was grown in egg yolk (yolk), tryptic soy broth (TSB), or grown in TSB followed by the administration of sterile egg yolk (TSB+Yolk). **(A)** Lethal dose 50 (LD_50_) of various treatment groups; data from the two independent repeats were combined to calculate LD_50_; error bar stands for standard error. **(B)** Survival curves for mice challenged with *Salmonella* Enteritidis; data from the two independent repeats (at all *Salmonella* inoculation levels) were combined. Statistical significance: (ns) not significant, *p* ≥ 0.05; (***) *p* < 0.001; (****) *p* < 0.0001.

Mice were fed, through oral gavage, with *Salmonella* Enteritidis that had been grown in egg yolk (yolk group), grown in TSB (TSB group), or grown in TSB with subsequent administration of sterile egg yolk (TSB+yolk group), and results are shown in **Figure 1A**. Significantly different (*p* = 0.0094) LD_50_ values were observed among these three groups. The LD_50_ of *Salmonella* Enteritidis was the lowest (2.8×10^2^ CFU) in mice of the yolk group, compared to that in the TSB group (1.1×10^3^ CFU) or the TSB+yolk group (4.6×10^3^ CFU). It is apparent from these results that growth in egg yolk, but not mere presence of the pathogen in sterile yolk in the gut, increased the virulence of *Salmonella* Enteritidis.

Mice survival curves representing individual *Salmonella* Enteritidis doses (10^2^ to 10^7^ CFU), pre-grown in egg yolk or TSB, were constructed (**Supplementary File S1**). Based on these curves, mice in the yolk group met the ERC faster, particularly at the higher *Salmonella* Enteritidis doses, compared to mice in two other groups. Survival curves for the three mice groups, combined at all dose levels, were constructed and compared statistically (**Figure 1B**). Mice survival curve for the yolk group was significantly different than that for the TSB group (*p* = 0.0002) or TSB+yolk group (*p* < 0.0001). However, survival curves corresponding to TSB and TSB+yolk mice groups were not significantly different (*p* = 0.987). The median survival ratio and hazard ratio for mice given *Salmonella* in yolk vs. TSB, calculated using Mantel-Haenszel model group, were 0.89 and 1.57, respectively.

### 3.2 Expression of Virulence Determinants When *Salmonella* Enteritidis Was Grown in Egg Yolk

To determine how virulence of *Salmonella* Enteritidis in mice might be enhanced by pre-growth in egg yolk, expression of selected virulence genes was compared when the pathogen was cultured in egg yolk and the microbiological medium, TSB. This was preceded with an experiment to determine the optimum phase of *Salmonella* growth for completing this analysis.

#### 3.2.1 Characteristics of *Salmonella* Enteritidis Growth in Egg Yolk

**Figure 2:**
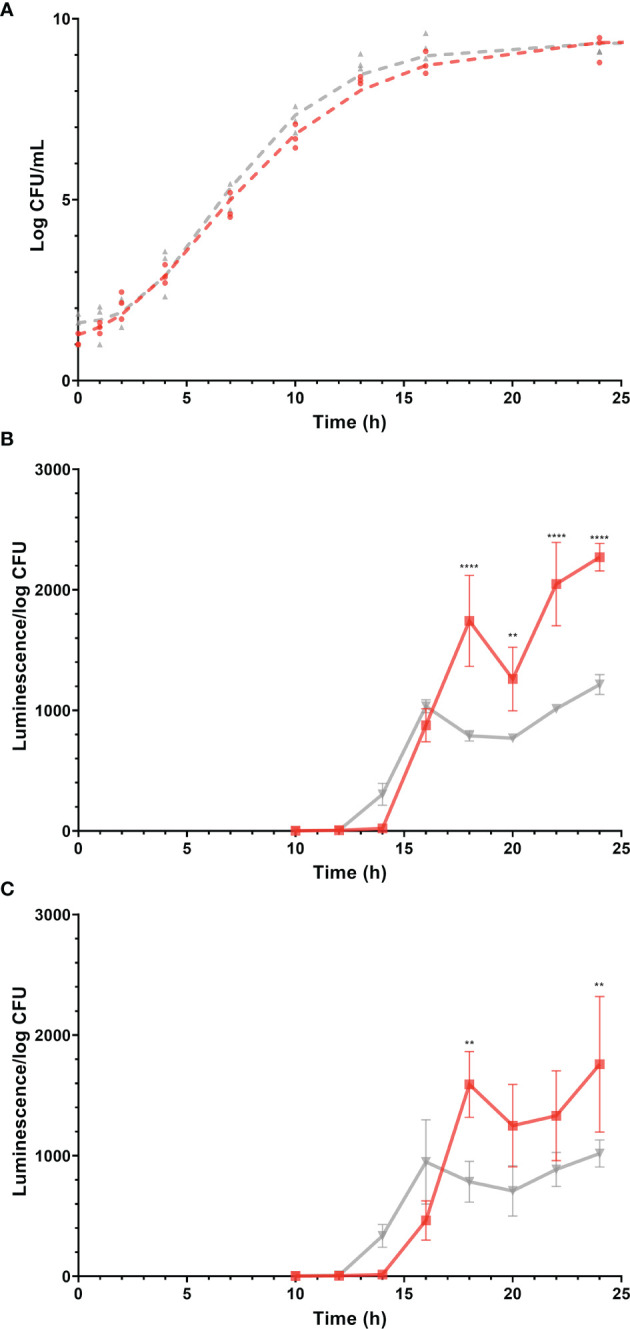
Growth (log CFU/mL) of *Salmonella* Enteritidis and expression (luminescence units/log CFU) of promoters of virulence-related genes, *sopB* and *sseA* during growth of the corresponding *Salmonella* Enteritidis reporter strains, in tryptic soy broth (TSB) and egg yolk. Gray and red lines (and symbols) represent TSB and yolk, respectively. Log CFU/mL values are represented by symbols and growth curves, simulated by the mathematical model, are shown as dashed lines. Changes in luminescence/log CFU are represented by solid lines. **(A)** Growth curves of *Salmonella* Enteritidis in egg yolk and in TSB. **(B)** Expression of *sopB*. **(C)** Expression of *sseA*. Each error bar represents the standard deviation for the average of three independent replicates. Statistical significance: (**) *p* < 0.01; (****) *p* < 0.0001.

The stage of growth is known to have a profound effect on gene expression in bacteria (Klumpp et al., 2009). To compare the expression of virulence genes of *Salmonella* Enteritidis in egg yolk and TSB, it was important to culture the pathogen in both media to the same stage of growth. Monitoring the growth of *Salmonella* Enteritidis in these media and modeling the resulting curves (**Figure 2A**) helped to define the time points suitable for harvesting RNA under these two different conditions. The predicted growth model in the TSB was


$$N=1.6+7.8\;e^{-e^{-0.30\times \left(t-6.0\right)}}$$

and in the yolk, it was


$$N=1.1+8.4\;e^{-e^{-0.23\times \left(t-5.9\right)}}$$

where N is *Salmonella* Enteritidis population (log CFU/mL) and *t* is the incubation time in hours. The performance measure, r^2^, for both models was 0.99, indicating a good fit of data by the model. The parallelism F-test and Chi-square test suggested that the overall shape of the two modeled growth curves was not significantly different (*p* > 0.05). However, when different stages of growth in both curves were compared, the predicted growth parameters for *Salmonella* Enteritidis in TSB and egg yolk were as follow: populations at lag phase were 1.6 ± 0.2 and 1.1 ± 0.2 log CFU/mL, populations at stationary phase were 9.4 ± 0.1 and 9.5 ± 0.2 log CFU/mL, growth rates were 0.30 ± 0.02 and 0.23 ± 0.02 log CFU/ml/h, and curve inflection points were 6.0 ± 0.3 and 5.9 ± 0.4 h, respectively. Based on these growth curves, it took *Salmonella* Enteritidis 13.0 h and 14.9 h to grow from < 10^2^ CFU/mL to 1.5 × 10^8^ CFU/mL in TSB and egg yolk, respectively. Preliminary data confirmed that the *Salmonella* Enteritidis population must reach 10^8^ CFU/mL, at least, to produce RNA with acceptable quality and quantity for conducting the transcriptional analysis. Therefore, the two time points mentioned previously (13.0 h and 14.9 h for incubation in TSB and egg yolk, respectively) were selected for harvesting cells to be used in the transcriptional analysis.

#### 3.2.2 Expression of Selected Virulence Genes at Various Growth Stages

*Salmonella* Enteritidis *sopB* and *sseA* reporter strains were grown in TSB or egg yolk and expression levels of *sopB* and *sseA* were used to indicate changes in the pathogen’s virulence capability. The expression of *sopB* and *sseA* was significantly higher when *Salmonella* Enteritidis was grown in egg yolk (*p* = 0.0019 and *p* = 0.0306, respectively), compared to its growth in TSB (**Figures 2B, C**). The expression of the virulence genes, *sopB* and *sseA*, was measurable after incubation of *Salmonella* Enteritidis for 12 to 14 h and 14 to 16 h in TSB and yolk, respectively (**Figure 2**) and both time ranges were within the late exponential phase of growth. The difference in lag times, between the start of incubation and the detection of expression, was possibly related to the fact that the late exponential phase came later in yolk than in TSB (**Figure 2**).

#### 3.2.3 Global Transcriptomic Analysis

**Figure 3:**
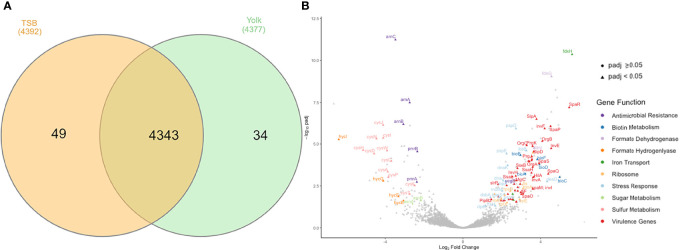
Genes transcribed by *Salmonella* Enteritidis, grown in tryptic soy broth or egg yolk, as revealed by RNA sequencing. **(A)** Summary of transcribed genes; **(B)** Volcano plot of differential expression results for all genes analyzed by RNA sequencing, emphasizing important differentially expressed genes.

RNA-seq data provided a global overview of the differences in gene expression between *Salmonella* Enteritidis grown in egg yolk and TSB. The total number of transcribed genes in both growth media was 4343 while only 34 and 49 genes were expressed solely when the pathogen grew in yolk and TSB, respectively (**Figure 3A**). Of the 4343 transcribed genes, 204 were differentially transcribed (*P*adj < 0.05); these included 74 downregulated and 130 upregulated genes (**Figure 3B**). Among the upregulated genes, the two largest groups were involved in virulence and stress response associated with membrane stress, respectively (**Figure 3B**). For instance, *baeS*, one of the membrane-stress regulators (Baron et al., 2017), and *ftsH*, encoding a metallopeptidase to maintain membrane protein integrity were significantly upregulated (**Figure 3B**). The phage shock protein (Psp) operon, which contains a set of genes induced under dissipation of proton motive force due to the impaired inner membrane integrity (Engl et al., 2011), and multiple heat stress response genes (*dnaK, dnaJ, clpB, ibpB*, and *ibpA*) that are commonly involved in bacterial membrane repair, as the cell envelope is the main target of heat injury (Aljarallah and Adams, 2007; Hews et al., 2019), were also upregulated (**Figure 3B**). *MutM*, which is involved in base excision repair of DNA damaged under stressed condition, was also included in the set of DEGs (**Figure 3B**). The formate dehydrogenase system (*fdnG*, *fdnI*, *fdoI*, and *fdhE*) that utilizes formate as an electron donor to regenerate proton motive force by forming redox loop, was upregulated, whereas the formate hydrogenlyase complex (*hycI*, *hycG* and *hycF*), which oxidizes formate to CO_2_ with the concomitant reduction of protons to H_2_, was repressed (**Figure 3B**) (Jormakka et al., 2002; Huang et al., 2008). Another set of genes among the downregulated DEGs included the *arn* operon and the regulator of the operon, *pmrAB* (**Figure 3B**). Those genes are related to the antimicrobial resistance against positively charged antimicrobial peptides, such as polymyxin, by masking the negative charge of the cell membrane and resulting in a more hydrophobic cell membrane (Huang et al., 2020; Park et al., 2021).

#### 3.2.4 Functional Enrichment and Pathway Analysis of Differentially Expressed Genes (DEGs)

**Figure 4:**
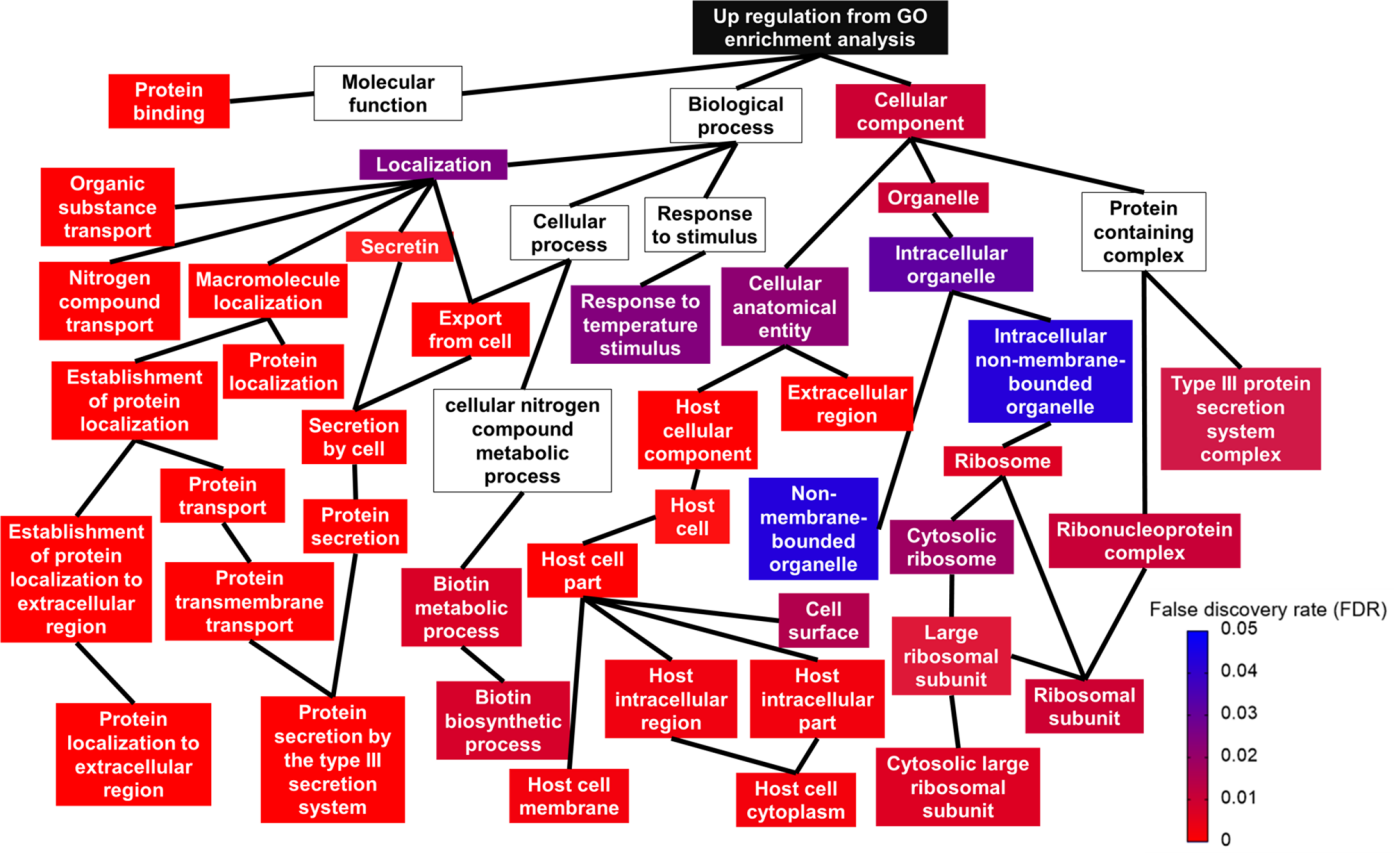
Relationship between upregulated gene ontology (GO) terms shown in a topologic structure. Each GO term is represented by a box. The color of the box (from red to blue) is an indication of false discovery rate; a box without any color means the GO term was not significant.

Using gene ontology (GO), DEGs were functionally analyzed to identify enriched functional categories in the *Salmonella* Enteritidis transcriptome. Significantly enriched terms in each GO category are displayed in **Figure 4** and **Supplementary File S2**. Among the biological categories, the upregulated DEGs were enriched in biotin biosynthetic processes and protein transport and localization, such as secretion by type III secretion system and protein localization to extracellular region (**Figure 4**). DEGs encoding type III secretion apparatus included *sipB, sipC*, *sipD*, *invA*, *invJ*, *prgH, prgI, prgK*, *spaO, spaP, spaR, spaS*, *orgA*, and *orgB* on SPI-1 and *sseB*, *sseC*, *ssaG*, and *ssaN* on SPI-2 while DEGs encoding effectors secreted through the type III secretion apparatus included *sopB*, *sopE*, and *sopE2* on SPI-1, *sopD2*, *sseL* on SPI-2, and *pipB* and *pipB2* on SPI-5. Sulfur compound metabolic processes, including hydrogen sulfide biosynthesis, were the most represented in the downregulated DEGs (**Supplementary File S2**). The enriched molecular function GO terms included protein binding in the upregulated DEGs (**Figure 4**), and sulfate transporter activity in the downregulated DEGs (**Supplementary File S2**). No downregulated DEGs were found significant by the enriched cellular component GO terms but for the upregulated DEGs, ribosome and type III secretion system-related cellular components were enriched (**Figure 4** and **Supplementary File S2**).

**Figure 5:**
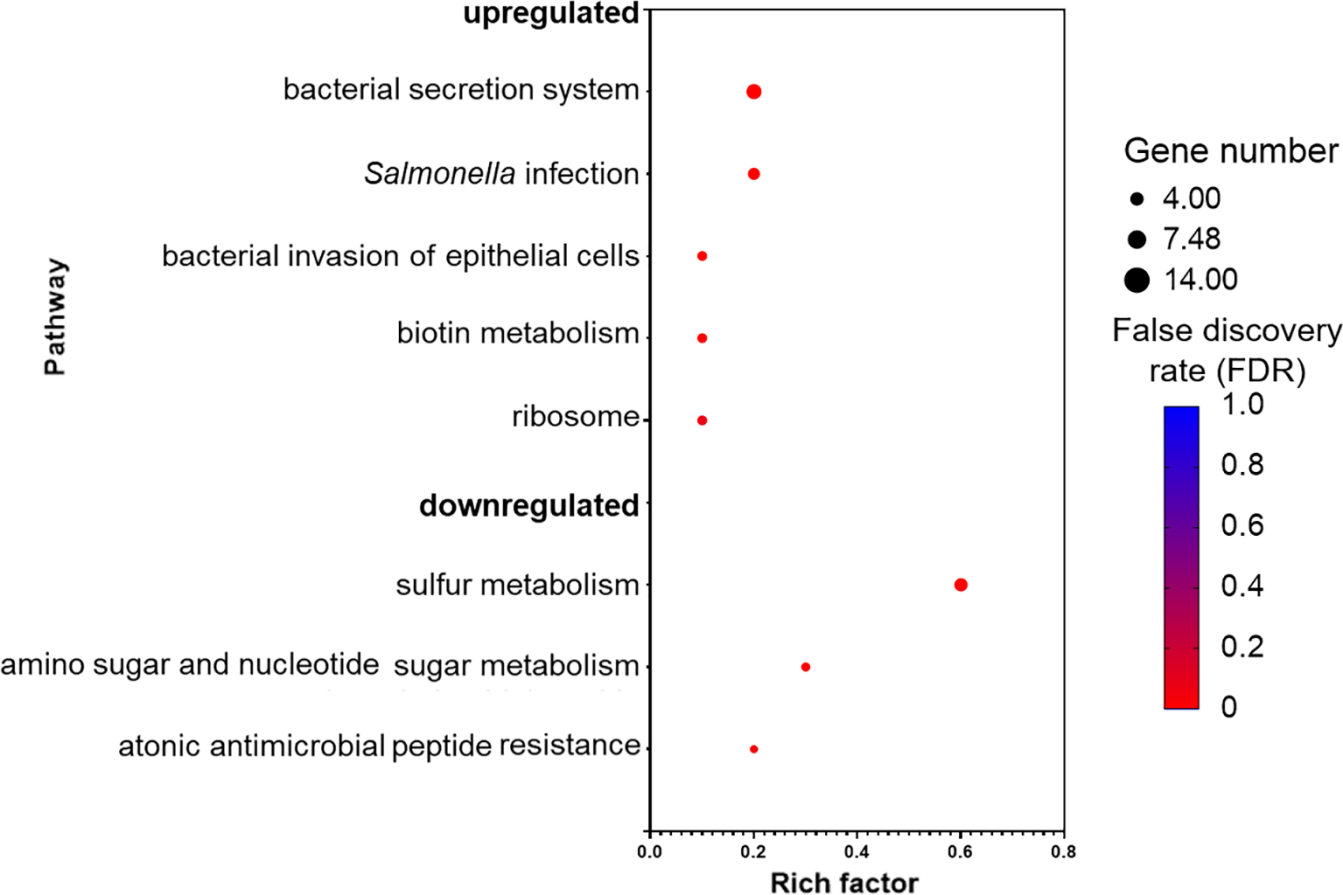
Pathways that are upregulated and downregulated as analyzed by Kyoto Encyclopedia of Genes and Genomes (KEGG). Each pathway is represented by a dot; the size, color, and rich factor value of the dot represent the total number of the genes within the pathway, false discovery rate, and proportion of genes in the pathway considered as core enrichment, respectively.

Kyoto encyclopedia of genes and genomes (KEGG) database mapping revealed several significantly enriched pathways (**Figure 5**). The majority of the enriched pathways in the upregulated DEGs were related to *Salmonella* virulence factors, such as “bacterial secretion system,” “*Salmonella* infection,” and “bacterial invasion of epithelial cells,” whereas three pathways were enriched in the downregulated DEGs and those were associated with sulfur and amino sugar metabolism, and antimicrobial peptide resistance.

**Figure 6:**
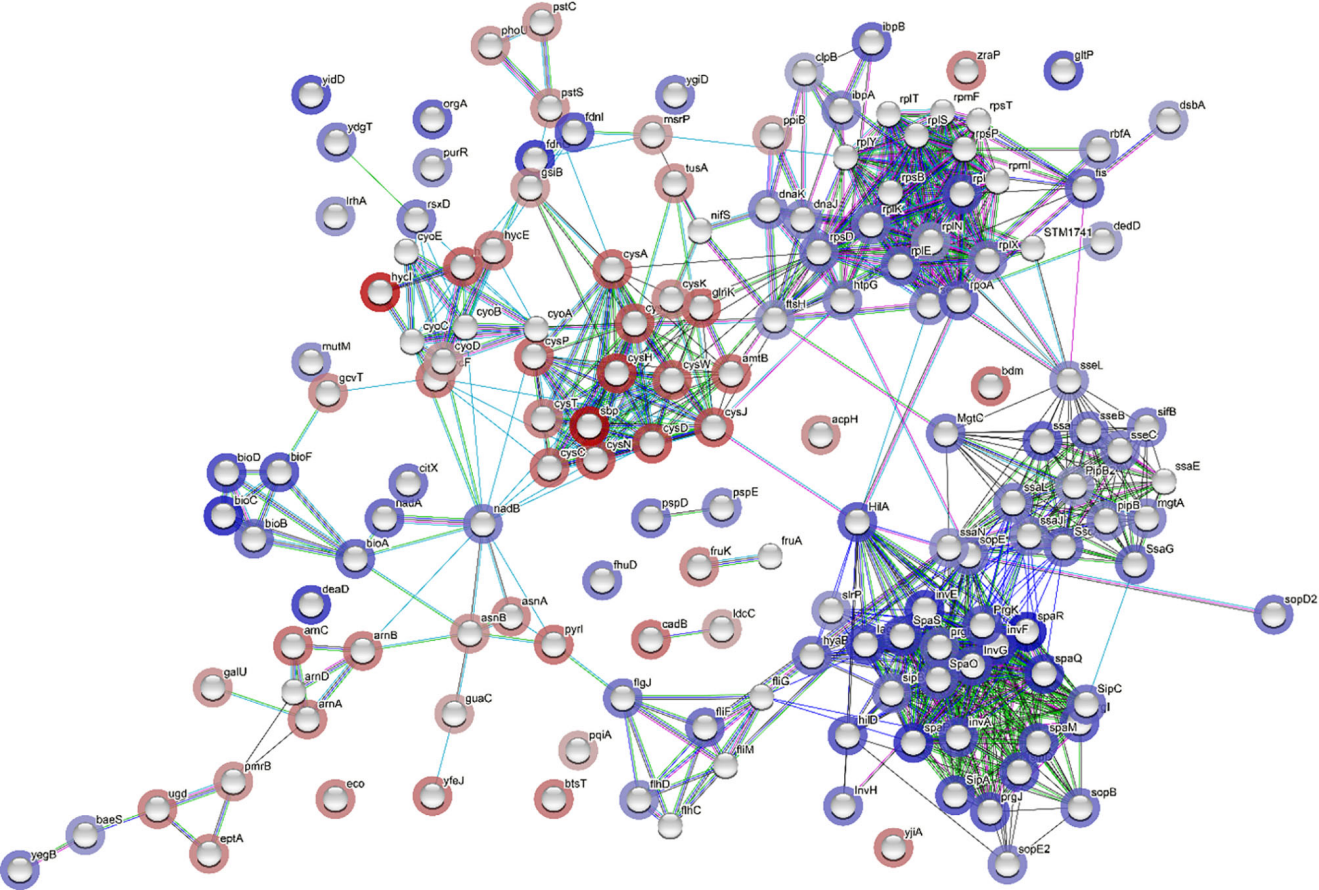
Network analysis through STRING showing correlation between all differentially expressed genes (DEGs). Each network node represents the gene matched with a DEG. The color of the surrounding halo indicates the rank of the gene among all the DEGs, based on fold changes; blue means upregulated while red, downregulated. The interactions between the genes are marked by lines. Each color of the lines suggests a source of evidence to the association. Turquoise means known interactions from curated databases; pink means experimentally determined known interactions; green, red and dark blue means predicted interaction from gene neighborhood, gene fusions, and gene co-occurrence, respectively; yellow, black and purple indicates information was obtained from text-mining, co-expression, and protein homology, respectively.

Network analysis was constructed for the DEGs using STRING tool (**Figure 6**). In the network analysis, functionally related genes are clustered together; this is obvious considering that DEGs encoding *Salmonella* infection process and type III secretion system (e.g., *sipA, sipB, sipC, sipD*, and *sopB*), and *Salmonella* invasion (e.g., *invA, ssaG, ssaJ, ssaN*, and *spaO*) were connected (**Figure 6**). The main connector in the network is *hilA*, which is a regulator gene for SPI-1 (**Figure 6**). It was noticed that *htpG*, a gene encoding heat shock protein, was co-expressed with other stress response genes (e.g., *dnaJ, dnaK, ibpB, clpB*) (**Figure 6**). Moreover, *htpG* interacts with virulence genes *via sopE*, which encodes an effector protein on SPI-1. The ribosome biosynthetic protein genes (e.g., *rplE, rplK, rplN, rplQ, rplX, rpsD*) are connected to *Salmonella* virulence genes *via sseL*, which encodes an effector protein, or *via rpoA*, which encodes RNA polymerase, and lastly connected to the protein translocase gene *secA*, which is involved in the translocation of *Salmonella* effector/invasion proteins into the host cell (**Figure 6**). Overall, the network analysis indicates that the transcription of *Salmonella* virulence genes was enriched and co-expressed with some stress response genes. The elevated expression of all these genes requires the ribosomal machinery to be upregulated.

#### 3.2.5 Gene Set Enrichment Analysis

**Figure 7:**
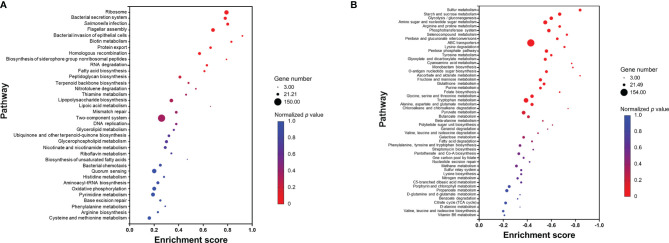
Pathways that are upregulated **(A)** or downregulated **(B)** based on gene set enrichment analysis (GSEA). Each pathway is represented by a dot; the size, color, and enrichment score value of the dot represents the total number of genes within the pathway, normalized *p* value, and proportion of the genes in the pathway considered as core enrichment, respectively.

In contrast to GO enrichment and KEGG analysis, gene set enrichment analysis (GSEA) identifies patterns of up- or downregulation of pre-defined gene sets from ranked lists of all analyzed genes. Among 87 defined gene sets (based on KEGG pathways), 11 were significantly upregulated and 24 significantly downregulated (**Figure 7**). The significantly upregulated pathways included ribosome, *Salmonella* virulence related pathways (including bacterial secretion system, *salmonella* infection, flagellar assembly, bacterial invasion of epithelial cells, and protein export), biotin metabolism, homologous recombination, biosynthesis of siderophore group non-ribosomal peptides, RNA degradation, and fatty acid biosynthesis (**Figure 7A**). A set of core enriched genes involved in “homologous recombination,” a pathway important to DNA damage repair, included *ssb1, recA, recF, recG, recN, recO*, *recR, ruvA*, *ruvC, dnaE*, *dnaX, holB-D*, *priA*, and *priB*. Within the significantly downregulated pathways, the majority are involved in sulfur, carbohydrate, and amino acid metabolism (**Figure 7B**).

#### 3.2.6 Verification of RNA-Seq Results Using RT-qPCR

**Figure 8:**
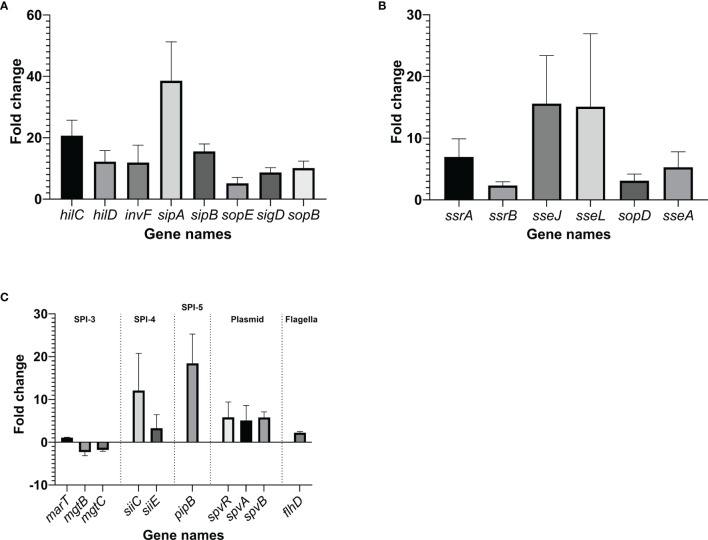
Changes in the transcription of virulence genes within *Salmonella* pathogenicity island (SPI)-1 **(A)**, SPI-2 **(B)** and other virulence components **(C)**, when the pathogen was grown in yolk compared to its growth in tryptic soy broth. The fold change was calculated through ΔΔCt method using *gapA*, a housekeeping gene, as a reference. Error bars represent standard deviation of three independent repeats.

RT-qPCR was performed on selected *Salmonella* virulence-related genes, using glyceraldehyde-3-phosphate dehydrogenase A gene, *gapA*, as a reference. When *Salmonella* Enteritidis was grown in egg yolk, genes tested within its pathogenicity islands SPI-1, SPI-2, SPI-4, and SPI-5, were significantly upregulated (>2-fold), compared to genes from cells grown in the TSB (**Figure 8**). Genes of SPI-3 (*mgtBC*) were downregulated and the genes *spvR* and *spvB* of *Salmonella* virulence plasmid were upregulated in the yolk (**Figure 8C**). Additionally, *flhD*, a gene that encodes the regulator for flagellar biosynthesis [which indirectly regulates SPI-1 genes *via* activation of the flagellar gene *fliZ* (Fabrega and Vila, 2013)], was also upregulated (**Figure 8C**). Within SPI-1, major regulators (*hilC* and *hilD*), a transcription regulator of type III secretion system (*invF*), as well as effector genes involved in bacterial internalization (*sipA* and *sipB*) (Fabrega and Vila, 2013) were highly upregulated (>10-fold). *SopB*, co-regulated with SPI1 and the protein product of which is secreted by type III secretion system on SPI-1, was also strongly upregulated. The major regulators of SPI-2 (*ssrA* and *ssrB*) were significantly upregulated but not as highly transcribed as the major regulators in SPI-1; however, *sseJ* and *sseL*, the two effector genes functioning to change host cell physiology and enhance bacterial survival in host cell (Uchiya et al., 2004; Uchiya and Nikai, 2004; Arena et al., 2011; Jennings et al., 2017) were noticeably upregulated. SPI-5 genes encoding for PipB, which is involved in accumulation of lipid rafts and the alternation of phospholipid structure of host cell membrane (Subramanian and Qadri, 2006; Shivcharan et al., 2018), were also appreciably upregulated. *PipB* is co-regulated with SPI-2 and its protein product is secreted by type III secretion system on SPI-2. These results confirmed the observations from RNA-sequencing analysis.

## 4 Discussion

Based on the mouse study, *Salmonella* Enteritidis grown in egg yolk showed the infectious pattern of faster disease onset and lower infectious dose, in comparison to *Salmonella* grown in a common microbiological medium, TSB (**Figure 1B**). Other researchers also found that mice orally challenged with *Salmonella* Enteritidis in the yolk displayed a significantly higher degree of pathogen intestinal colonization, fecal shedding, and dissemination in liver and spleen, and inflammation of liver and ceca, compared to mice orally challenged with *Salmonella* Enteritidis in LB, another common microbiological medium (Moreau et al., 2016). In previous studies, researchers argued that food characteristics could have a protective effect for the pathogens. For example, high viscosity could delay gastric acid emptying, proteins may have a buffering effect against gastric acids, and fat might form a protective layer around the pathogens (Ponder, 2017). These proposed effects of the *Salmonella*-carrying medium cannot explain the results of the current study because mice challenged with *Salmonella* grown in TSB, which was immediately followed by administering sterile egg yolk, did not show the same disease severity as the mice challenged with *Salmonella* grown in egg yolk. Therefore, the increase in *Salmonella* virulence, as observed in the current study, cannot be attributed to the protective effect of the yolk medium.

### 4.1 *Salmonella* Enteritidis Grown in Egg Yolk Induced Virulence Genes Transcription

When *Salmonella* invades macrophage, SPI-2 plays a fundamental role in pathogen survival and cell-to-cell spread, whereas SPI-1 and flagella-related genes are strongly downregulated (Eriksson et al., 2003; Hautefort et al., 2008). However, when *Salmonella* invades epithelial cells, not only SPI-1 but SPI-2 and flagella are also strongly upregulated (Hautefort et al., 2008). In the current study, growth of *Salmonella* Enteritidis in egg yolk upregulated many genes including virulence genes in SPI-1, SPI-2 and flagella genes (**Figures 2**, **4**, **5**, and **7A**). Thus, it is likely that *Salmonella* spread and reproduce in egg yolk in a manner similar to that during its invasion of epithelial cells. The key metabolic status of *Salmonella* in egg yolk was in concert with the signature metabolic status of the pathogen during epithelial invasion. For instance, Hautefort et al. (2008) reported that when *Salmonella* is inside epithelial cells, its transcription of genes involved in biosynthesis of biotin (an enzyme cofactor for fatty acid biosynthesis) was induced 10-fold, compared to that for *Salmonella* inside a macrophage. Biotin biosynthesis was the top-ranked upregulated pathway in the current study (**Figure 7A**). Additionally, biosynthesis of siderophore was a significantly upregulated pathway (**Figure 7A**). Studies supported that siderophores are important for *Salmonella* fitness in the gastrointestinal tract (Pi et al., 2012; Saha et al., 2017; Saha et al., 2019).

Using network analysis, connections were observed among DEGs encoding ribosome biosynthetic proteins, *Salmonella* virulence proteins, and the translocation of *Salmonella* effector/invasion proteins into the host (**Figure 6**). This implied that *Salmonella* Enteritidis in egg yolk was programmed to actively produce virulence effector proteins through generous ribosomal activity even before the pathogen infects any animal host. During the intestinal internalization, pre-produced virulence effectors could assist rapid invasion and colonization in the host, whereas *Salmonella* from other sources may have to adapt to the host first, sense the signals from the host for transcription of virulence factors, and assemble virulence proteins before the invasion (Marcus et al., 2000; Hurley et al., 2014). Consequently, *Salmonella* Enteritidis grown in egg yolk is more apt to cause salmonellosis over the pathogen grown in other media, as supported by the current animal study results (**Figure 1**). With such remarkable protein secretion events, active metabolic pattern of *Salmonella* is required for combating the draining of bacterial energy levels (Hautefort et al., 2008). According to GSEA analysis (**Figure 7A**), upregulated metabolic pathways in egg yolk included nitrotoluene degradation, thiamine metabolism, glycerolipid metabolism, and lipoic acid metabolism. It is still unclear if these metabolic pathways worked together to support such a high level of energy demand or serve the essential metabolic needs that allow energy-efficient proliferation of *Salmonella* in the egg yolk.

### 4.2 Increased Virulence of *Salmonella* Enteritidis in Egg Yolk Is Potentially Related to Stress Responses Assisting Membrane and DNA Repair

The constructed network for DEGs using STRING analysis associated upregulated virulence factors to membrane-related stress response (**Figure 6**). This membrane stress can be related to the impaired inner membrane integrity leading to dissipation of proton motive force, consistent with the upregulation of the *psp* operon (Engl et al., 2011). To restore the proton motive force, *Salmonella* in egg yolk potentially utilized formate dehydrogenase system to regenerate protons and suppress the function of formate hydrogenlyase that transforms protons to H_2_ (**Figure 3B**). Researchers have linked *psp* operon not only to heat shock response (Jovanovic et al., 2006) which was within the upregulated DEGs (**Figure 3B**), but also to virulence (Eriksson et al., 2003; Jovanovic et al., 2006; Faucher et al., 2006; Hautefort et al., 2008; Karlinsey et al., 2010; Wallrodt et al., 2013; Wallrodt et al., 2014). For example, the *psp* system is believed to be important for uptake, survival, and replication of *Salmonella* in macrophages (Eriksson et al., 2003; Faucher et al., 2006; Hautefort et al., 2008; Wallrodt et al., 2014). Beside these membrane stress related genes, genes involved in homologous recombination for DNA repair were also strongly upregulated (**Figure 7A**). It seems that *Salmonella* Enteritidis in egg yolk experience stress, which requires DNA and bacterial membrane repair. This also matched with the observation that it took *Salmonella* in egg yolk approximately 2 additional hours to enter the growth exponential phase, in comparison to *Salmonella* grown in TSB (**Figure 2A**). It seems that the upregulated cascade of DNA repair mechanisms reflects massive nucleotide alterations when *Salmonella* grew in egg yolk.

### 4.3 *Salmonella* Enteritidis Encounters Stresses in Egg Yolk Environment

Significant down regulation of nutrient metabolism, e.g., glycolysis, by *Salmonella* Enteritidis in egg yolk was observed (**Figure 7B**). It is possible that egg yolk sugars (e.g., glucose) interact tightly with the hydrophilic head of yolk phospholipids by forming hydrogen bonding (Pereira and Hünenberger, 2006); this interaction could limit the availability of this metabolizable sugar to *Salmonella*. Additionally, the largest fraction of egg yolk protein, lipovitellins, is non-covalently linked to nearly all the lipids at its large hydrophobic binding cavity forming non-water-soluble yolk granule (Hetta, 2008), limiting the availability of the protein source to *Salmonella*. Wang et al. (2021) showed that genes in the phage shock protein (*psp* family), DNA repair proteins, and heat stress response chaperones were upregulated during starvation stress of *Escherichia coli* incubated in saline for 24 h.

According to a preliminary experiment, viscosity of egg yolk was 2.1 Pa·s, which is equivalent to that of corn syrup, and egg yolk was 40 times more viscous than TSB. This high viscosity may have hindered the mobility of *Salmonella* and its nutrient uptake ability. In order to compensate for the limited movement in highly viscous medium, *Salmonella* could switch from swimming state (movement through liquids) to swarming state, a hyperflagellated state of bacterial cells allowing extra motility (Wang et al., 2004). This agrees with our finding of upregulation of flagella related genes when *Salmonella* was grown in egg yolk (**Figures 6** and **7A**). Additionally, *Salmonella psp* response is commonly accompanied with swarming (Wang et al., 2004; Erhardt and Dersch, 2015). In the context of virulence, the increased flagellar biosynthesis accelerates the ability of the pathogen to approach epithelial cells during the adhesion stage (Stecher et al., 2004). As a matter of fact, in *Enterobacter* spp., flagellar development is often associated with the disruption of the Cyc protein family, which are related to sulfate reduction (Sturgill et al., 2004; Rossi et al., 2014; Hufnagel et al., 2018; Westerman et al., 2021); genes of this protein family were the most profoundly repressed group in the current study (**Figure 7B**). In order to maximize flagellar rotation during the swarming stage, *Salmonella* has to maintain a hydrated shell around the cell by altering its cell membrane structure significantly to decrease the hydrophobicity of the LPS (Toguchi et al., 2000). The downregulation of *arn* operon could possibly serve the function of producing a more hydrophobic LPS.

## 5 Conclusion

The current study demonstrated that growth in egg yolk significantly enhanced the virulence of *Salmonella* Enteritidis; this likely contributes to the risk of salmonellosis associated with consumption of contaminated eggs. Once grown in yolk to the late exponential phase, *Salmonella* not only replicated rapidly but also induced transcription of virulence factors. Therefore, the same pathogenic strain could show different pathogenicity in different food vehicles. Hence, the food vehicles that carry pathogens should be given considerable importance in foodborne disease assessment models and in disease mitigation policies. This study also identified potential mechanisms underlying the upregulation of *Salmonella* virulence in eggs. However, further work is needed to test the mechanisms proposed in this study.

## Data Availability Statement 

The original contributions presented in the study are included in the article/**Supplementary Material**. Further inquiries can be directed to the corresponding author. The RNA sequencing data are publicly available in National Centre for Biotechnology Information (NCBI) sequence read archive (SRA) under accession number SRX14788580 and SRX14788579 for yolk and TSB, respectively. 

## Ethics Statement

The animal study was reviewed and approved by the Ohio State University Institutional Animal Care and Use Committee (Protocol OSU 2009A0035).

## Author Contributions

The contribution of authors to the article in each item is listed in the order of last name. This article was conceptualized by AA, YX, and AY. BA and AY cooperated on funding acquisition. Experimental planning and methodology design were collaborated between AA, BA, AS-D, and YX. Experiments were executed by AA, AS-D, and YX while data curation was conducted by AA and YX. Formal data analysis was contributed by AA, MS, and YX. The original draft was written by AA and YX while reviewed and edited by AA, BA, AS-D, MS, YX, and AY. The entire process was supervised by BA and AY while the project was administrated, and resources were provided by AY. All authors contributed to the article and approved the submitted version.

## Funding

This work is supported by the USDA National Institute of Food and Agriculture, AFRI project 2020-67017-30794.

## Conflict of Interest

The authors declare that the research was conducted in the absence of any commercial or financial relationships that could be construed as a potential conflict of interest.

## Publisher’s Note

All claims expressed in this article are solely those of the authors and do not necessarily represent those of their affiliated organizations, or those of the publisher, the editors and the reviewers. Any product that may be evaluated in this article, or claim that may be made by its manufacturer, is not guaranteed or endorsed by the publisher.
