# Supplementary material for: The Role of Egg Yolk in Modulating the Virulence of Salmonella Enterica Serovar Enteritidis
Source: Front Cell Infect Microbiol. 2022 Jun 14;12:903979. doi: 10.3389/fcimb.2022.903979 (PMC9237210; doi:10.3389/fcimb.2022.903979)
Supplement: Supplementary file 1 [file DataSheet_1.docx]

Supplementary Material

# Supplementary Figures

**Supplementary Figure 1.** Mice survival curves by dose of administered *Salmonella* Enteritidis ranging from 10^7^ **(panel A)**, 10^6^ **(panel B)**, 10^5^ **(panel C)**, 10^4^ **(panel D)**, 10^3^ **(panel E)**, and 10^2^ **(panel F)** CFU.
