# Supplementary figures and images for: The Role of Egg Yolk in Modulating the Virulence of Salmonella Enterica Serovar Enteritidis
Source: Front Cell Infect Microbiol. 2022 Jun 14;12:903979. doi: 10.3389/fcimb.2022.903979 (PMC9237210; doi:10.3389/fcimb.2022.903979)

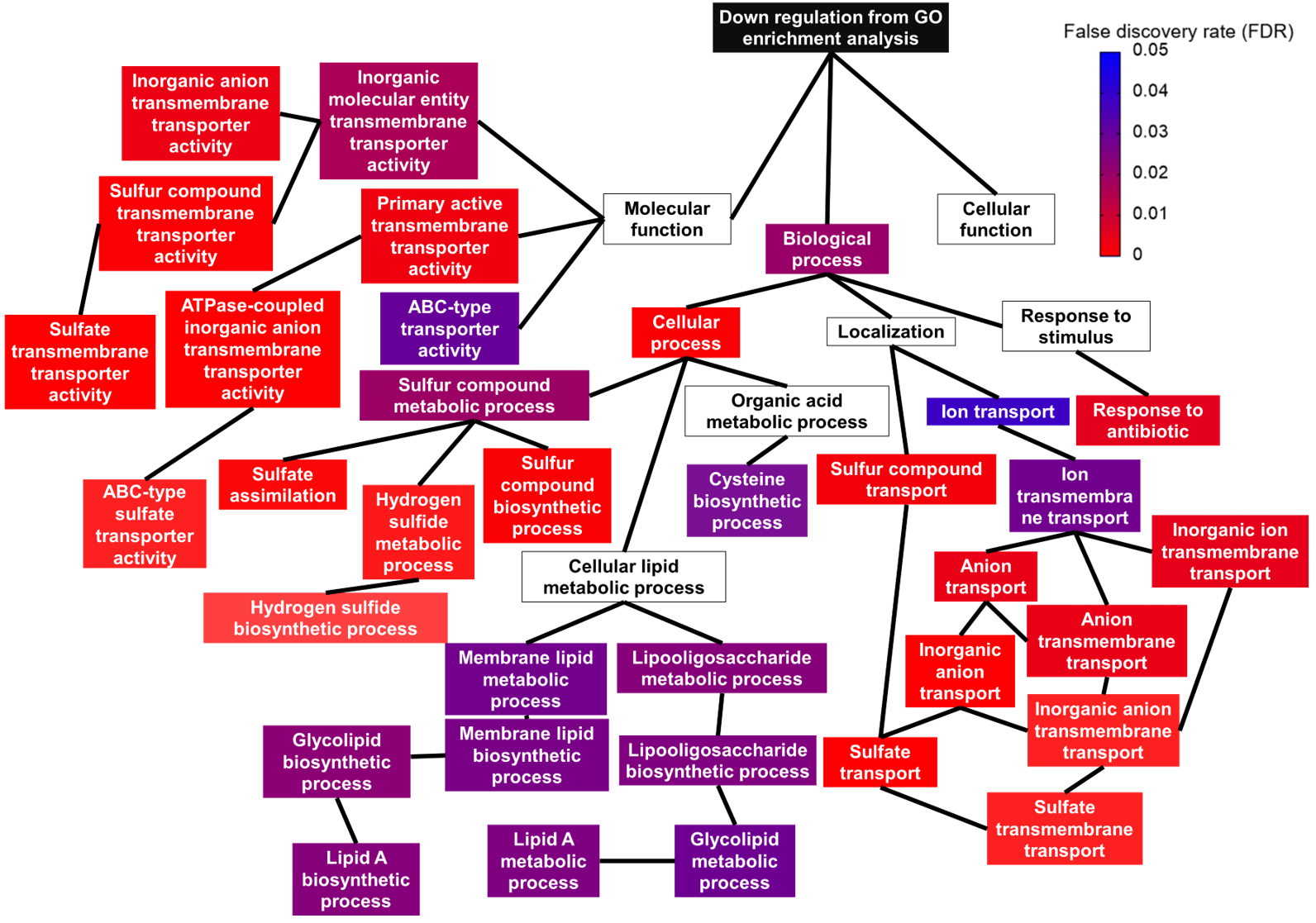

Supplement: Supplementary file 2 [file Image_1.png]
